# Pelleted-hay alfalfa feed increases sheep wether weight gain and rumen bacterial richness over loose-hay alfalfa feed

**DOI:** 10.1371/journal.pone.0215797

**Published:** 2019-06-05

**Authors:** Suzanne L. Ishaq, Medora M. Lachman, Benjamin A. Wenner, Amy Baeza, Molly Butler, Emily Gates, Sarah Olivo, Julie Buono Geddes, Patrick Hatfield, Carl J. Yeoman

**Affiliations:** 1 Biology and the Built Environment Center, University of Oregon, Eugene, Oregon, United States of America; 2 Department of Animal and Range Sciences, Montana State University, Bozeman, Montana, United States of America; 3 Department of Animal Sciences, The Ohio State University, Columbus, Ohio, United States of America; University of Wisconsin Madison, UNITED STATES

## Abstract

Diet composed of smaller particles can improve feed intake, digestibility, and animal growth or health, but in ruminant species can reduce rumination and buffering–the loss of which may inhibit fermentation and digestibility. However, the explicit effect of particle size on the rumen microbiota remains untested, despite their crucial role in digestion. We evaluated the effects of reduced particle size on rumen microbiota by feeding long-stem (loose) alfalfa hay compared to a ground and pelleted version of the same alfalfa in yearling sheep wethers during a two-week experimental period. *In situ* digestibility of the pelleted diet was greater at 48 h compared with loose hay; however, distribution of residual fecal particle sizes in sheep did not differ between the dietary treatments at any time point (day 7 or 14). Both average daily gain and feed efficiency were greater for the wethers consuming the pelleted diet. Observed bacterial richness was very low at the end of the adaptation period and increased over the course of the study, suggesting the rumen bacterial community was still in flux after two weeks of adaptation. The pelleted-hay diet group had a greater increase in bacterial richness, including common fibrolytic rumen inhabitants. The pelleted diet was positively associated with several *Succiniclasticum*, a Prevotella, and uncultured taxa in the Ruminococcaceae and Rickenellaceae families and Bacteroidales order. Pelleting an alfalfa hay diet for sheep does shift the rumen microbiome, though the interplay of diet particle size, retention and gastrointestinal transit time, microbial fermentative and hydrolytic activity, and host growth or health is still largely unexplored.

## Introduction

It has been well established that the nutrient composition of a diet affects the gastrointestinal tract (GIT) microbiota [1,2], yet the physical structure and complexity of the diet may also alter its interactions with the GIT microbiota and host. In cattle, longer fiber particles have been shown to improve rumination [3] and ultimately fiber digestibility [4]. However, shorter or smaller diet particles can change the dynamics of digestion and produce a number of favorable outcomes. Specifically, reductions in particle size associated with mastication or mechanized processing correspond to increases in feed surface area and thus allow for greater microbial attachment and relative fibrolytic and fermentative activities as has been shown *in vitro* [5]. Mechanical breakdown during diet preparation can also physically disrupt waxy plant cuticles and cell walls that can otherwise impede microbial attachment and degradation, thus making plant carbohydrates more available [6] and decreasing the potential confounding effect of forage fragility within the rumen [7]. Hydrolytic activities are also likely further enhanced by the reductions in buoyancy and increased functional specific gravity [8] associated with reduced particle size, that would allow smaller particles to sink beneath the dorsally-located rumen mat and into the microbe-rich rumen liquor [9,10].

Particle size not only affects the amount of surface area available for microbial attachment [11], but also the retention time of feed within the rumen environment. Feed has been shown to remain confined to the rumen until it reaches a critical size threshold–demonstrated by a number of studies to range from ~1 to 1.18 mm [12–14] after which it can escape the filtering capacity of the omasum [3,15–17]. Consequently, reducing the particle size of ruminant diets has been seen to increase GIT passage rate [18,19] and can thereby free up additional capacity for rumen fill which encourages dry matter intake (DMI). Both increased DMI and increases in hydrogen gas partial pressure that can result from rapid fermentation can further increase GIT passage rate and reduce ruminal retention time independent of particle size [12–14]. After exiting the rumen via the omasum, digesta proceeds to the highly acidic abomasum wherein microbial activity is largely halted [20]. Thus, the increased potential for fibrolytic activity associated with reductions in feed particle size are offset by the shorter time available for these activities to occur. Within this context, the effect of feed particle size on rumen microbial fibrolytic activity remains largely unexplored.

Additional factors that can further complicate the relationship of feed particle size and digestibility include the knowledge that ingesting smaller particles reduces the amount of time spent chewing [15,21–23] and ruminating [3,16,17]. Further, less mastication and rumination decreases the amount of the buffered saliva being swallowed [21,24], and as saliva is responsible for neutralizing 30 to 50% of the acid resulting from fermentation [25,26] this can lead to acidification of the rumen [25], and associated reductions in microbial attachment and fibrolytic activity [27–29]. Bacterial ionic charge is facilitated by cell walls composition; negatively-charged bacteria are better able to attach to lignocellulosic material, often mediated through binding to positively-charged magnesium [30]. The downstream impact on animal performance is further complicated. The solubility of dietary nutrients are also altered by the physics of particle size, thus affecting their ease of host digestion, including transit through mucus, and absorption by epithelia [11,31]. Similarly, small or homogenous particle sizes have been reported to preclude normal papillae development [32,33], although these alterations have been either reported to be localized within the GIT [33] or not significantly altered [34–36]. Other studies reported that roughage decreased omasal size and epithelial keratinization [34], and increased rumen wall muscularity over starch-based diets [35,36]. Lignin, cellulose, and hemicellulose can form superstructures in the luminal digesta, making digestible carbohydrates less accessible, and further contributing to nutrient loss through the binding of metal ions [30]. Thus, decreasing particle size has been demonstrated to affect the lignin content of feed by physical disruption [22], and can improve access to fiber and microbial fermentation *in vitro* and *in vivo* [22,23,37–39]. Overall the influence of particle size on animal performance, measured as feed efficiency or weight gain, is unclear with several studies having reported improvements with decreased particle size [23,32,40] while others do not [41–43]. These differences are likely the result of varied rumen conditions imposed by reduced particle size or other confounding conditions.

The objective of this research was to determine the effect of particle size reduction on animal growth, feed intake, feed digestibility, and microbial diversity by comparing lambs fed either a long-stem second-cutting alfalfa hay or the same alfalfa after pelleting. Our working hypothesis was that decreasing particle size of the alfalfa hay and delivery in the form of a pellet would increase animal growth and feed digestibility due to reduced particle size. Further, pelleted feed was hypothesized to decrease microbial diversity by allowing for more rapid fermentation and alteration of environmental conditions in the rumen.

## Materials and methods

### Ethics statement

All animal procedures were approved by the Montana State University Agricultural Animal Care and Use Committee (Protocol #2014-AA04). The trial was conducted at the Montana State University Bozeman Agriculture Research and Teaching Farm in Bozeman, MT, USA.

### Diet

Second cutting alfalfa was harvested and hayed in fall 2014 and used to produce 2 dietary treatments. The first treatment consisted of the loose, long-stem alfalfa hay, and the second treatment comprised the same hay being commercially ground and pelleted. Nutritional profiles of each diet were determined via NIR by Dairy One Forage Lab (Ithaca, NY) and are provided in Table 1. While it was intended to have the diets balanced for NDF, ADF, CP, and ME, the mechanical grinding and pelleting process induced some unavoidable changes to the forage provided that resulted in minor differences between diets among some nutrient categories. Since the pellets were derived from the same hay source, the primary contributing factors to variation between treatments was likely shatter of leafy particles from hay at grinding and pelleting, and the addition of some oil to properly bind the pellet. Fiber (NDF and ADF) were decreased by the pelleted diet while CP was increased.

**Table 1:** Nutritional composition of loose-hay and pelleted-hay alfalfa diets, as well as *in situ* rumen digestibility.

	Loose feed	Loose refusals	Pelleted feed	Pellet refusals
Moisture	8.6	7.9	10.2	9.4
Dry Matter (DM; %)	91.4	92.1	89.8	90.6
Crude Protein (CP; % DM)	16	13	20	20
Non-Fiber Carbohydrates (NFC)	18.2	16.8	22.5	23.2
Neutral-Detergent Fiber (NDF; % DM)	54.6	59.7	46.8	46.2
peNDF_1.18_ (% DM)	44.3		14.4	
Acid-Detergent Fiber (ADF; % DM)	43.8	49.1	37.9	37.5
Lignin (% DM)	10.8	11.8	9.3	9
Ash (% DM)	6.9	5.8	10.4	9.9
Total Digestible Nutrients (TDN)	52	49	58	58
Relative Feed Value (RFV)	93	79	118	120
% Digested 24 hr *in situ* (*P =* ns)	50.36%		56.30%	
% Digested 48 hr *in situ* (*P < 0*.*001*)	51.50%		58.80%	

### Experimental design

Yearling Rambouillet wethers (n = 10, avg. BW = 30 ± 5 kg) were randomly assigned to either loose-hay or pelleted diets (n = 5) within blocks by weight. Animals were adapted to a 50:50 mix of both dietary treatments over 14 d prior to the start of the trial, with the final 7 d being spent in metabolism crates with fastened fecal bags for acclimation. The end of the adaptation period was considered time point zero (day 0/week 0), following which the animals were fed their assigned diet for a 14-day period. Animals were housed indoors in metabolism crates during the trial period in an uninsulated barn that lacked temperature control. Temperatures averaged 13° C with 63% humidity during the trial period and lights were cycled on for 12 h and off for 12 h. Wethers were fed *ad libitum* twice daily between the times of 0700 to 0830 and 1730 to 1900. Orts and feces were collected; water was available free choice at all times. Animals were weighed once weekly, and blood and rumen samples collected at the start of the trial (day 0, end of adaptation period) and at days 7 and 14. A total of 50 ml rumen samples were collected between the hours of 14:00 and 16:00 using sterilized foal tubes with a 600cc large animal dosing syringes, and immediately stored at -20°C until processing (within 3 weeks). Feed samples were randomly taken in triplicate daily from hay bales using a Penn State Forage Sampler (loose) or pellet bags (pellet) and pooled by treatment day to submit a single feed sample for laboratory analysis. Following weighing, feed samples and orts were stored in paper bags at ambient temperature until grinding. Feces were collected and frozen at -20°C to prevent microbial metabolism.

#### Intake and digestibility

Intake was determined as feed provided less orts. Dry matter (DM) of feed, orts, and feces was determined by drying overnight in an oven set to 100°C. Digestibility of alfalfa treatments was estimated by 48 h *in situ* (i.e. *in sacco*) incubation in a rumen-cannulated cross-bred Angus heifer using a standardized procedure as previously described [44]. Particle size distributions of feed, orts, and feces averaged over 3-day blocks were determined by wet sieving as previously described [45] using a set of 12 mesh sieves (Sigma Aldrich, St. Louis, MO, USA) ranging from No. 4 (> 4.76mm) down to No. 200 (0.074–0.087 mm). Using lignin as an internal marker, fecal nutrient composition was used to predict apparent nutrient digestibility (DM, CP, NDF, ADF) using the equation [46]:

$$Apparent\;digestibility=100-[100\;x\;\left(\frac{{lignin}_{diet}}{{lignin}_{feces}}\right)x\;\left(\frac{{nutrient}_{feces}}{{nutrient}_{diet}}\right)]$$

Means for sheep intake and digestibility were compared in a mixed effects model (SAS 9.4, Cary, NC) with fixed effect of treatment, random effect of sheep, and a repeated sampling day. Repeated covariance structure was selected using the lowest BIC and significance was declared at *P* < 0.05. Means for sheep average daily gain (ADG) and feed conversion ratio (FCR; kg gain/ kg DMI) used the same structure with the removal of repeated measures since there were only 3 weekly samples included. Means for *in situ* digestibility were compared via linear mixed modeling (lmer) and least squares means (lsmeans [47]), with sheep as a fixed effect to control for repeated measures, and significance was designated at *P* < 0.05 with tendencies from 0.05 *≤ P* < 0.10.

### Blood panel

Blood samples (10 mL) were collected from each wether by jugular venipuncture. All samples were collected using serum vacutainers (Becton Dickinson, Franklin Lakes, NJ) fitted with 20-gauge vacutainer needles. Serum tubes were allowed to coagulate before being placed on ice and transported to the Montana Veterinary Diagnostic Laboratory (Bozeman, MT, USA) where large animal biochemistry panels were performed using an automated biochemical analyzer. The panel included creatine kinase/phosphokinase (CPK), creatinine (CREAT), aspartate aminotransferase (AST), alkaline phosphatase (ALKP), total protein (TP), albumin (ALB), globulin (GLOB), glucose (GLU), blood urea nitrogen (BUN), calcium (Ca), phosphorus (P), sodium (Na), potassium (K), chloride (Cl), magnesium (Mg), total bilirubin (T Bili), direct bilirubin (D Bili), and total carbon dioxide (TCO2). Means for blood measurements (S1 Table) were compared in a mixed effects model (SAS 9.4, Cary, NC) with fixed effect of treatment, random effect of sheep, and a repeated sampling day. Repeated covariance structure was selected using the lowest BIC and significance (unadjusted) was declared at *P* < 0.05 with tendencies from 0.05 *≤ P* < 0.10.

### Bacterial V3-V4 16S rRNA profiles

Rumen samples were thawed on ice, and bulk nucleic acids extracted via the MoBio PowerFecal DNA Isolation kit (Carlsbad, CA, USA) following manufacturer protocols. PCR amplification, amplicon quantification and purification, and Illumina MiSeq sequencing were performed as previously described [48] using the V3-V4 region of the 16S rRNA gene using a 300-cycle kit. Forward sequences were processed using the DADA2 package [49] in R [50], using filterAndTrim command parameters: trimLeft = 10, truncLen = 270, maxN = 0, maxEE = 2, truncQ = 2, rm.phix = TRUE. Sequences had taxonomy assigned with the Silva ver. 132 taxonomy database, sequences identified as chloroplast or mitochondrial were removed, as were chimeras. Negative controls included at DNA extraction through to sequencing were used to remove sequences from samples which also appeared in negative controls, as per (https://github.com/SueIshaq/Examples-DADA2-Phyloseq). Workflow was validated using an in-house 32 member mock community, as previously described [51]. Per sample, the number of sequences which passed quality-control measures ranged from 3,520–221,441, and for comparison, sequence variants (SVs) were subsampled down to 3,520 per sample. Remaining sequences were analyzed using packages phyloseq [52], DESeq [53], rfPermute [54], and visualized with ggplot2 [55]. Observed richness and Shannon diversity values were determined to not be normally distributed via a Shapiro-Wilks Test. Observed richness was analyzed using a generalized linear mixed-effects model (GLMM) with lme4 [56], using Sheep ID as a fixed effect. Shannon diversity created a singular model fit, and was analyzed with a Conover Test for paired comparisons of non-normal data; Kruskal-Wallis chi-squared = 20.329, degrees of freedom = 5, model *P* < 0.001. Importance variables were identified using distance-based redundancy analysis (dbRDA; capscale) and variance partitioning (varpart). Community distance was assessed using unweighted Jaccard distance and adonis (permanova) in vegan [57], with Sheep ID as a fixed effect. Significance was designated at *p* < 0.05. Raw sequence reads are available from NCBI SRA under BioProject Accession number PRJNA384590.

## Results

### Intake, particle size, digestibility

**Fig 1:**
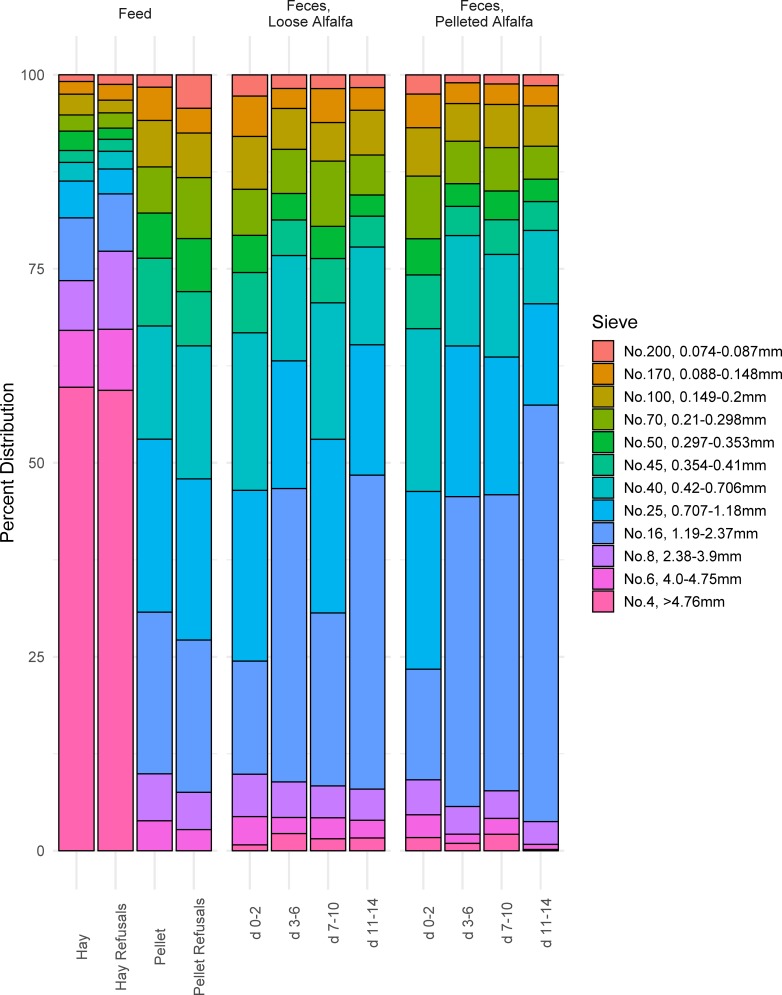
Particle size distribution of in diet treatment biomass, and percentage of residual feed particles in feces in wethers on a loose-hay (Hay) and pelleted (Pellet) alfalfa diet. Intake was averaged across 3-day blocks over a 14-day period.

The two diets differed in their particle size distributions (Fig 1). Loose-hay alfalfa stems were an average 109.8 ± 44.9 mm in length, and 59.7% of hay particles were retained above the 4.76 mm screen (largest sieve used) while the majority (57.7%) of pelleted alfalfa feed was between 0.42 and 2.37 mm. Importantly, 18.4% and 69.2% of mass of the two diets, respectively, were less than the 1.18 mm threshold previously reported [12–14] to be the critical size for feed to escape the rumen. Calculated peNDF_1.18_ [58] of the loose hay and pelleted diets were 44.3% and 14.4%, respectively. Particles from hay refusals (orts) averaged 95.6 mm ± 60.1 in length, thus, we concluded that sorting against large particles did not occur in the loose hay diet. Following consumption and digestion, distribution of residual fecal particle sizes did not differ between diets at any time point sampled (Fig 1, S2 Table; *P* > 0.05).

Nutritional analyses of the two diet treatments are shown in Table 1. *In situ* dry matter disappearance at 48 h indicated that the pelleted diet was more digestible than the whole hay diet (58.8% compared to 51.5%, *P* < 0.001).

Feed intake was affected by rumen and blood serum sample collections on days 6 and 14 with both groups showing a reduction, and so these data were removed from further analysis (S3 Table). Overall, feed intake was significantly greater (*P* = 0.01) for wethers fed the pelleted diet compared with the loose hay diet; average dry matter intake increased by 17% (1.60 vs 1.86 kg/d, respectively) (Table 2). Although low for both groups, average daily gain (ADG) throughout the trial was greater (*P* = 0.0003) for wethers consuming the pelleted diet (0.24 +/- 0.02 kg/d) than for the loose hay diet (0.08 +/- 0.02 kg/d). The difference in ADG between pelleted and loose hay treatments was numerically greater after week 2 (*P* = 0.0003, 0.312 and 0.143 kg/d, respectively) than in the first week (*P* = 0.02, 0.169 and 0.026 kg/d, respectively). Although feed intake was increased in the pelleted diet, feed conversion ratio (FCR) was significantly (*P =* ≤ 0.02) improved in week 1 (0.079 vs 0.013), week 2 (0.196 vs 0.121), and overall (0.129 vs 0.053) for pelleted versus loose hay diets, respectively. Estimated apparent NDF and ADF digestion using lignin as an internal marker was greater (*P* ≤ 0.004) for sheep fed loose hay than for sheep fed the pelleted diet (Table 2). In contrast, apparent CP digestibility was greater (*P* < 0.0001) in sheep fed the pelleted diet than the loose hay diet.

**Table 2:** Weight, intake, and efficiency values for wethers on loose-hay diet and pelleted-hay alfalfa diets.

	Loose Hay	Pelleted	SE	P-value
Feed Intake (kg/d)	1.6	1.86	0.06	0.01
Average Daily Gain (kg/d)	0.084	0.24	0.018	0.0003
Week 1	0.026	0.169	0.036	0.02
Week 2	0.143	0.312	0.019	0.0003
Feed Conversion Ratio	0.0527	0.129	0.008	0.0002
Week 1	0.0128	0.0786	0.0167	0.02
Week 2	0.121	0.196	0.014	0.006
Apparent Digestibilties				
App DMd	35.6	40.7	2.2	0.12
App CPd	38.4	57.5	2.4	<0.0001
App NDFd	34.9	26.7	1.8	0.004
App ADFd	38.4	30.5	0.8	<0.0001

Apparent digestibility was estimated using lignin as an internal marker.

#### Blood parameters

Serum values are provided in Table 3, including creatine kinase/phosphokinase (CPK), creatinine (CREAT), aspartate aminotransferase (AST), alkaline phosphatase (ALKP), total protein (TP), albumin (ALB), globulin (GLOB), glucose (GLU), blood urea nitrogen (BUN), calcium (Ca), phosphorus (P), sodium (Na), potassium (K), chloride (Cl), magnesium (Mg), total bilirubin (T Bili), direct bilirubin (D Bili), and total carbon dioxide (TCO2). Blood creatinine was not different between treatments (*P* = 0.18) but CPK tended to be increased in the pelleted treatment by 135 units/L (Table 3; *P* = 0.06). Total protein showed a tendency to be greater in the pelleted diet (*P* = 0.08) but there were no differences in albumin nor globulin measurements to explain the variation (*P* ≥ 0.20). Many other standard panel measurements (AST, ALKP, GLU, and both total and direct bilirubin were unaffected by treatment (*P* ≥ 0.37). Blood urea nitrogen was greater in both treatment groups than the normal reference range for sheep [59] but pelleted treatment sheep were also greater than loose hay fed sheep (*P* = 0.02) by 4,25 mg/dL. Blood Ca was slightly greater in the pelleted treatment (*P* = 0.05) while P was decreased by 1.5 mg/dL in the pelleted diet (*P* = 0.0008). Finally, total blood CO_2_ was decreased within the pelleted treatment group but both treatment estimates are well within the range of normal for this test.

**Table 3:** Blood serum parameters for wethers fed either a loose alfalfa hay or pelleted alfalfa diet.

Parameter^1^	Loose Hay	Pelleted	SE	*P*-value
CPK	299.49	434.64	43.06	0.06
CREAT	0.682	0.79	0.052	0.18
AST	113.99	114.73	7.93	0.95
ALKP	192.39	200.4	17.38	0.75
T_PRO	6.49	6.88	0.14	0.08
ALB	3.1	3.08	0.06	0.76
GLOB	3.27	3.51	0.12	0.2
GLU	63.62	66.68	2.43	0.4
BUN	27.7	31.95	1.08	0.02
Ca	10.29	10.52	0.07	0.05
P	7.48	5.97	0.2	0.0008
Na	143.92	142.92	0.36	0.09
K	4.7	4.82	0.12	0.48
Cl	104.41	104.66	0.34	0.61
Mg	2.27	2.27	0.05	0.97
T_BILI	0.406	0.42	0.031	0.75
D_BILI	0.07443	0.08721	0.009603	0.37
CO_2_	28.36	24.58	1.02	0.03
HEM_Index	0.278	0.234	0.048	0.54

^1^Blood parameter abbreviations are as follows: CPK: creatine phosphokinase, CREAT: creatinine, AST: aspartate aminotransferase, ALKP: alkaline phosphatase, T_PRO: total protein, ALB: albumin, GLOB: globulin, GLU: glucose, BUN: blood urea nitrogen, T_BILI: total bilirubin, D_BILI: direct bilirubin, HEM Index: index of blood hemolysis.

### Bacterial richness in lamb rumen

**Fig 2:**
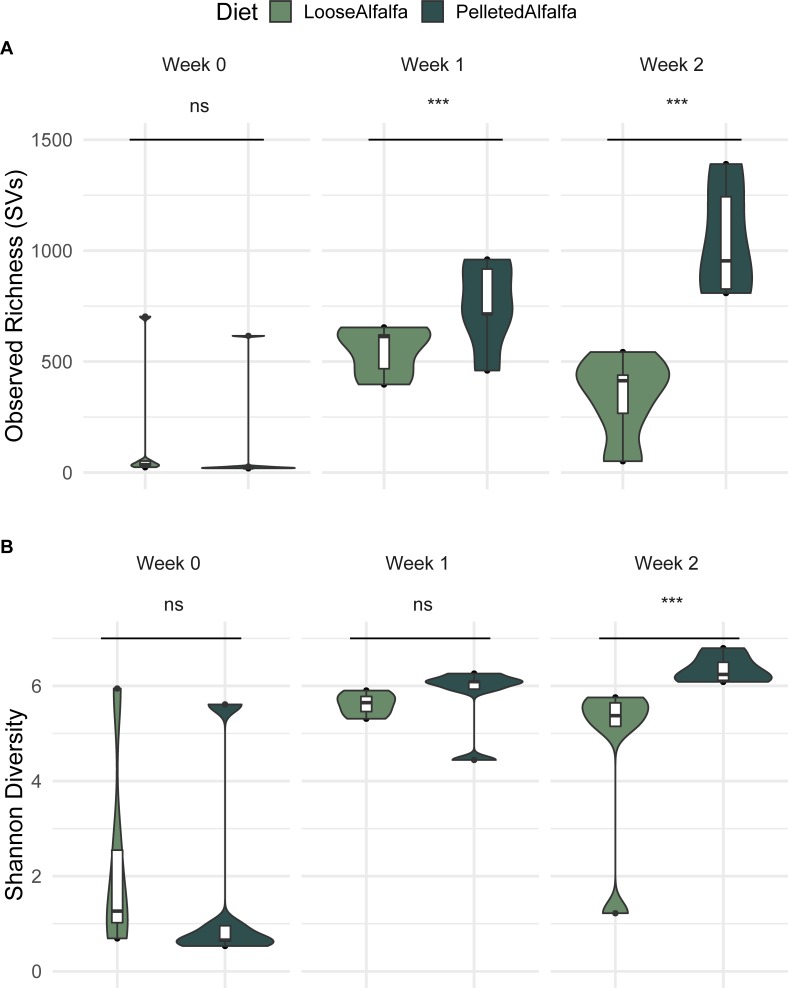
**Observed bacterial richness (A) and Shannon diversity (B) in the rumen of wethers on loose-hay or pelleted-hay alfalfa diets.** Significance was determined at *p* < 0.05, by linear mixed model for observed SVs and Conover test for Shannon diversity, with sheep ID as a fixed effect.

**Fig 3:**
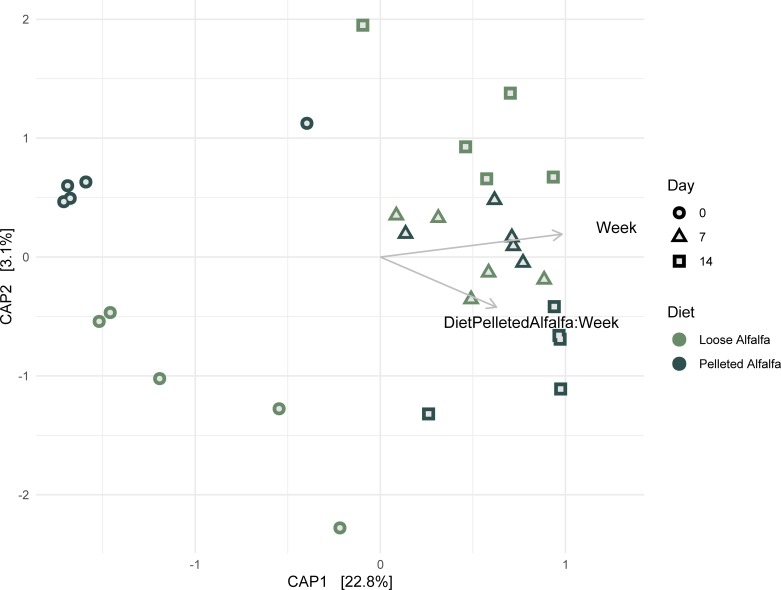
Distance-based redundancy analysis of Bray-Curtis Distance between rumen bacterial communities in wethers receiving loose-hay or pelleted-hay alfalfa diets.

Observed bacterial richness was very low in week 0, the end of the adaptation period (Fig 2A); it increased between days 0 and 7 (*P* < 2e-16), and days 0 and 14 (*P* < 2e-16), suggesting the rumen bacterial community was still in flux after two weeks of adaptation. Bacterial richness was significantly greater in the pelleted alfalfa diet at day 7 (*P* < 2e-16) and day 14 (P < 2e-16). Shannon diversity was significantly different between diets at day 14 (Fig 2B; *P* = 0.0025). Only day (F = 5.3686, *P* = 0.001) significantly explained overall bacterial community clustering in constrained ordination (Fig 3); diet and diet x day were not significant. Overall, the constrained model was significant (F = 3.154, *P* = 0.001). However, day did not significantly contribute to variation in the community (varpart, variance = 0%), though community diversity contributed to 60% and day x diversity contributed to 39% of variance.

**Fig 4:**
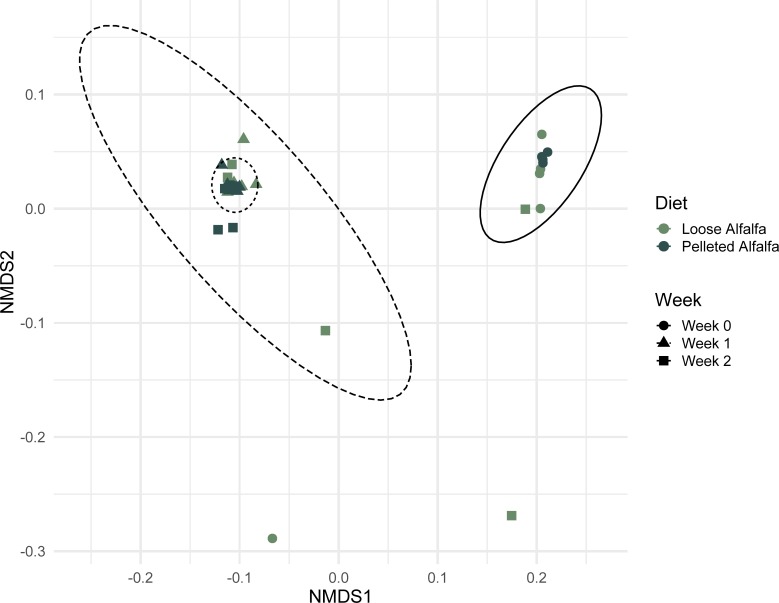
Non-metric multidimensional scaling (NMDS) plot of unweighted Jaccard distance between rumen bacterial communities in wethers receiving loose-hay or pelleted-hay alfalfa diets. Ellipses represent treatment weeks. Lowest stress = 0.0649, R^2^ = 0.9958.

Similarly, bacterial community composition clustered by sample day, followed by diet, in unconstrained ordinations (Fig 4). Despite the increased richness, there was no significant diet:day interaction between bacterial communities based on unweighted (comparing samples based on OTU presence or absence)β-diversity (*P* = 0.31), however there was an effect of sample day (F = 2.44, R^2^ = 0.15, *P* = 0.0001) and dietary treatment overall (F = 1.50, R^2^ = 0.05, *P* = 0.037). Weighted (comparing samples based on OTU abundances) bacterial community distance, using Bray-Curtis Dissimilarity, was only significantly different by sample day (F = 4.64, R^2^ = 0.25, *P* = 0.0001).

**Fig 5:**
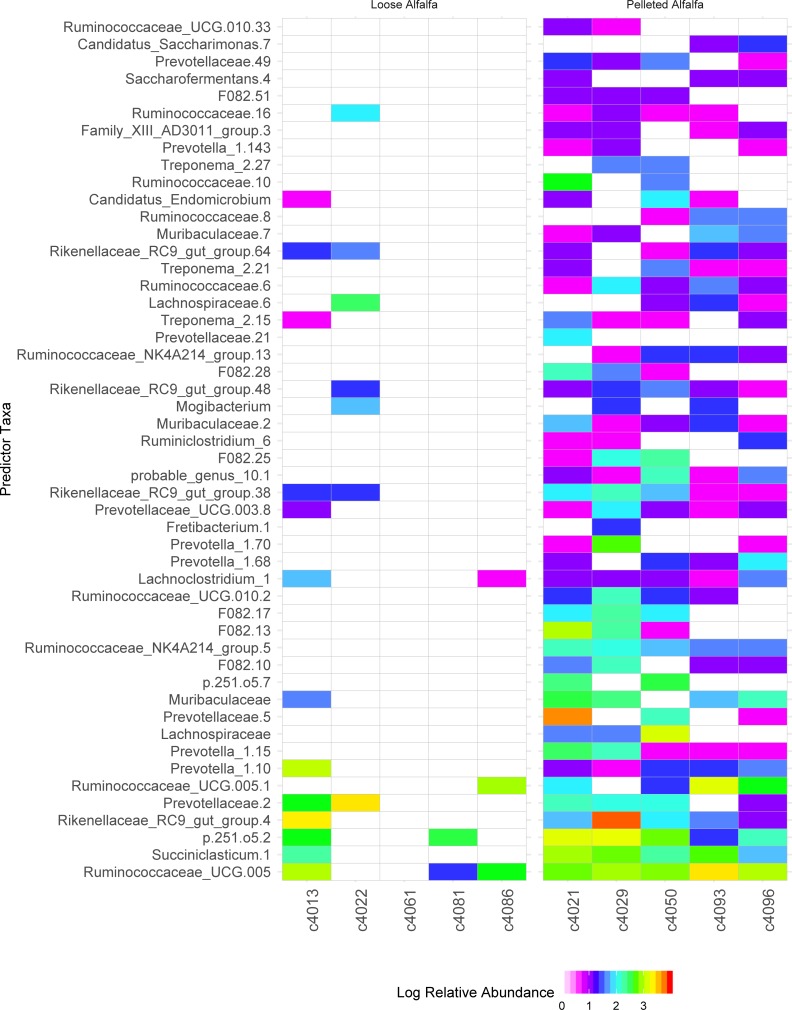
Discriminatory rumen bacterial sequence variance by treatment group for wethers receiving loose-hay or pelleted-hay alfalfa diet treatments. Significance (*p* < 0.05) determined by binomial test.

When examining samples from day 7 and 14, to minimize any potential impact of dietary adaptation occurring between trial days 0 and 7 samples, 86 SVs were significantly discriminatory to the pelleted diet over loose-hay alfalfa diets, the 50 most abundant of which are presented in Fig 5. These included Ruminococcaceae UCG-005, *Succiniclasticum*, the RC9 gut group, and several *Prevotella*. Random forest prediction accuracy for diet was 80%, using only days 7 and 14 for reference, but was only 60% accurate in determining discriminatory communities using all sample days (0, 7, and 14), despite the close clustering of samples observed in Fig 4. Likewise, just 30% accuracy was obtained in predicting bacterial communities for treatment x sample day.

### Correlation of bacterial richness to performance parameters

**Fig 6:**
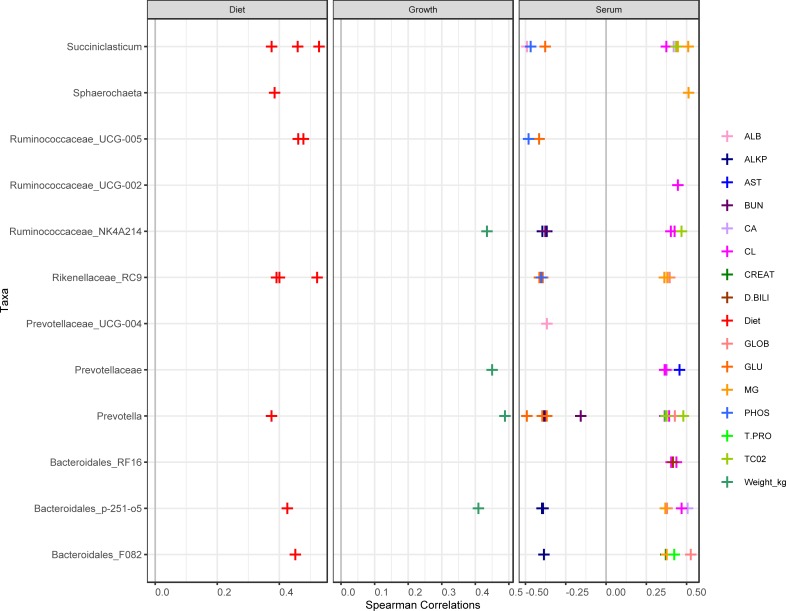
Correlations between the 50 most abundant rumen bacteria and lamb growth, serum parameters, and treatments. Only significant (*P* < 0.05) Spearman’s correlations are reported.

The 86 rumen bacterial sequence variants (SVs) which were significantly discriminatory between lamb wethers on loose-hay and pelleted-hay alfalfa diets, as identified using random forest, were corelated against lamb weight and blood serum chemistry (Fig 6). Spearman’s correlation revealed a number of bacterial taxa which were correlated with particular parameters.

A number of the most abundant bacterial taxa identified in the rumen were significantly correlated with lamb growth and health, as well as with diet treatment (Fig 6). Body weight was positively correlated with several SVs in Prevotellaceae family, an SV in the Ruminococcaceae family, and an uncultured bacterium in the Bacteroidales order (p-251-05). The pelleted diet was positively associated with several *Succiniclasticum*, a Prevotella, and uncultured taxa in the Ruminococcaceae and Rickenellaceae families and Bacteroidales order. Several serum factors were correlated with lamb body weight (Table 4).

**Table 4:** Serum factors significantly correlated (Spearman’s) with weight in lambs.

Serum Factor	R	p
CL	0.408037	0.025196
CPK	-0.3864	0.034931
CREAT	0.435603	0.016125
GLOB	0.373562	0.04201
T.BILI	-0.36713	0.045963
T.PRO	0.408708	0.024934

## Discussion

Despite the known effect of diet particle size on physical egress through the GIT, chemical accessibility and digestibility, and host absorption, the explicit effect of feed particle size has never been examined within the context of rumen microbial diversity. Differences in the structural fiber composition and resulting bioavailability of nutrients that occur among plant species can select for distinct microbial communities [60]. In the present study, pelletization of an alfalfa diet increased bacterial diversity and sheep performance, presumptively via a smaller particle size which thereby improving protein digestion of feed.

In other studies comparing the same diet across multiple particle sizes, no difference in ADG were observed by particle size regardless of an increase in DMI [42,43]. Although ADG was seen to differ in our study, both groups exhibited much lower ADG than National Research Council expectations [61], which may have been influenced by the short study period and artificial environment (metabolism crates) that the lambs were confined to, thus this result should be interpreted cautiously. Lambs fed the pelleted diet did out-consume lambs fed the loose hay. Likely, increased DMI decreased digestibility for lambs fed the ground and pelleted diet [62]. This loss in digestibility was anticipated given a wealth of previous literature on fiber particle reduction, feed intake response, and reduced digestibility [63]. However, loss in digestibility was offset by net increase in digestible energy supply which combined with improved apparent CP digestibility contributed to improved gain in the pelleted diet lambs. The improvement in feed conversion ratio for pellet fed lambs is simply explained as a dilution of maintenance, where lambs eating and digesting more feed will have additional energy and protein in excess of requirement to allocate towards growth.

Some particle reduction studies using cows report loss of efficiency of microbial protein synthesis and total diet digestibility, perhaps in part due to a poor functioning rumen mat [64]. A smaller particle increases the available surface area for microorganisms to attach to feed but also increases passage rate from the rumen; the difference between ground and unground forage would be exacerbated in cows where particle reduction by mastication is less than in sheep [37]. A faster passage rate may sacrifice some ruminal fiber digestibility [65] yet increase microbial protein synthesis efficiency as bacteria compete to subsist in more rapid turnover environments [66,67]. In the present study, increased residue of NDF and ADF relative to lignin in the feces of sheep fed the pelleted diet indicate a poorer digestibility of fiber when provided in a ground, pelleted form. Studies have shown a U-shaped relationship of lignin content in the residual material of feces to particle size [22], supporting the idea that an overly-fine diet may not be beneficial. Given the effect of decreasing particle size on rumen retention time [22], this loss in fiber digestibility observed in the present study was expected and pairs with foundational work on the effect of grinding on fiber digestibility [68]. Furthermore, mechanical processing and pelletization of feed, while creating more surface area, can also compact the open space created by complex carbohydrate structure, thereby reducing the water-holding capacity of the feed and increasing its density, which may have contributed to lower microbial digestion [37].

In the present study, most serum values were within normal ranges, with the exception of creatine phosphokinase (CPK) at week 1 for pelleted-hay sheep, which was high for some individuals [69]. This spike in CPK presumably led to the detected significance for pelleted treatment to increase CPK compared with the loose hay diet. Traditionally, CPK has been associated with selenium deficiency induced white muscle disease in sheep, high sulfur-induced polioencephalomalacia [70,71], or copper toxicity [72]. However, none of these explain the current treatment difference and it is possible the greater CPK values may reflect short-term sampling stress [73].Digestibility crates notoriously disrupt animal behavior; perhaps the strong flocking instinct of sheep created high stress levels for some individuals that was not reflected in feed intake data [59,73].

Blood urea nitrogen concentration in the present study was increased in the pelleted diet. The pelleted diet ground and thus more prone to greater (and estimated apparent) CP digestibility; increased BUN in the pelleted treatment supports this data as greater rumen degradable protein or (leading to ruminal ammonia concentration) in the diet will increase circulating urea recycling to the rumen [74]. The high concentration of BUN in both treatments indicates degradable protein was in excess for both treatments. More research needs to be done on the appropriate balance of degradable to undegradable protein for growing sheep in order to prevent systemic overfeeding of degradable protein in lambs.

Limited small ruminant studies do not report an effect of particle size per se on blood biochemical parameters in goats fed rice straw [75] or lambs fed alfalfa [35]; however, some studies do report an effect on some serum metabolites and protein digestion values by single bacterial species when introduced to gnotobiotic animals [76–79] A review of probiotic in lambs reported both increased and decreased levels of blood urea nitrogen, glucose, and creatine, citing changes in fermentation and ammonia cycling resulting from altered rumen bacterial diversity [80]. In the present study, blood urea nitrogen, calcium, creatine, globulin, magnesium, and total protein were positively correlated many bacterial taxa, while sodium, phosphorous, potassium, and total carbon dioxide were negatively correlated with many other bacteria.

Observed bacterial richness was very low in week 0 at the end of the adaptation period, and then increased for the rest of the study, suggesting the rumen bacterial community was still in flux even after two weeks of adaptation. Similarly, samples clustered largely by week, indicating the need for longer dietary adaptation periods in ruminant studies. The pelleted-hay diet recruited significantly more bacterial richness, including common rumen inhabitants known to ferment a variety of carbohydrates, such as *Prevotella*, *Ruminococcus*, *Succiniclasticum*, and members of the RC9 gut group. Yet despite the increase in average daily gain and feed efficiency, there was no difference in residual fecal nutrients by diet except protein, which was reduced in pelleted-hay lambs. It is possible that the improvement in animal performance was not from fiber digestion but from enhanced access to microbial and dietary protein resulting from a combination of greater increased microbial growth due to improved access to dietary nutrients making available more surplus energy for increasing microbial biomass, and an increased flow rate of digesta and associated microbiota from the rumen.

## Supporting information

S1 Table Serum parameters for wethers receiving either a loose-hay or a pelleted-hay alfalfa diet treatment for two weeks. (DOCX)

S2 Table Residual nutritional composition of feces from wethers on loose-hay or pelleted-hay alfalfa diets over the two-week experimental period. (DOCX)

S3 Table Daily intake by diet. (DOCX)

## References

[1] Henderson G, Cox F, Ganesh S, Jonker A, Young W, Global Rumen Census Collaborators, et al Rumen microbial community composition varies with diet and host, but a core microbiome is found across a wide geographical range. Sci Rep. 2015;5: 14567 10.1038/srep14567 26449758 PMC4598811

[2] David LA, Maurice CF, Carmody RN, Gootenberg DB, Button JE, Wolfe BE, et al Diet rapidly and reproducibly alters the human gut microbiome. Nature. 2014;505: 559–563. 10.1038/nature12820 24336217 PMC3957428

[3] Beauchemin KA. Invited review: Current perspectives on eating and rumination activity in dairy cows. J Dairy Sci. 2018;101: 4762–4784. 10.3168/jds.2017-13706 29627250

[4] Allen MS, Mertens DR. Evaluating Constraints on Fiber Digestion by Rumen Microbes. J Nutr. 1988;118: 261–270. 10.1093/jn/118.2.261 2828582

[5] Bjorndal KA, Bolten AB, Moore JE. Digestive Fermentation in Herbivores: Effect of Food Particle Size. Physiol Zool. University of Chicago Press; 1990;63: 710–721. 10.1086/physzool.63.4.30158172

[6] McAllister TA, Bae HD, Yanke LJ, Cheng K-J, Ha JK. A review of the microbial digestion of feed particles in the rumen. Asian-Australian J Anim Sci. 1994;7: 303–316.

[7] Cotanch K, Grant R, Miner WH. Fiber digestibility and forage fragility in dairy cattle. Cornell Nutrition Conference. Ithaca, NY; 2008.

[8] Siciliano-Jones J, Murphy MR. Specific Gravity of Various Feedstuffs as Affected by Particle Size and In Vitro Fermentation. J Dairy Sci. Elsevier; 1991;74: 896–901. 10.3168/jds.S0022-0302(91)78238-6

[9] Wattiaux MA, Satter LD, Mertens DR. Effect of microbial fermentation on functional specific gravity of small forage particles1. J Anim Sci. Narnia; 1992;70: 1262–1270. 10.2527/1992.7041262x 1582955

[10] Wattiaux MA, Mertens DR, Satter LD. Kinetics of hydration and effect of liquid uptake on specific gravity of small hay and silage particles. J Anim Sci. Narnia; 1992;70: 3597–3606. 10.2527/1992.70113597x 1459920

[11] McClements DJ, Xiao H. Potential biological fate of ingested nanoemulsions: influence of particle characteristics. Food Funct. The Royal Society of Chemistry; 2012;3: 202–220. 10.1039/c1fo10193e 22105669

[12] Domingue BMF, Dellow DW, Wilson PR, Barry TN. Comparitive nutrition of deer and goats, goats and sheep. Proc Ihe New Zeal Soc Anim Prod. 1990;50: 39–42.

[13] Oshita T, Nonaka K, Kume S, Nakui T. Effects of forage type on particle size distribution of ruminal digesta and faeces of non-lactating cows fed high quality forage. Livest Prod Sci. 2004;91: 107–115. 10.1016/j.livprodsci.2004.07.015

[14] Teimouri Yansari A, Valizadeh R, Naserian A, Christensen DA, Yu P, Eftekhari Shahroodi F. Effects of alfalfa particle size and specific gravity on chewing activity, digestibility, and performance of Holstein dairy cows. J Dairy Sci. 2004;87: 3912–3924. 10.3168/jds.S0022-0302(04)73530-4 15483175

[15] Grant RJ, Colenbrander VF, Albright JL. Effect of Particle Size of Forage and Rumen Cannulation upon Chewing Activity and Laterality in Dairy Cows. J Dairy Sci. Elsevier; 1990;73: 3158–3164. 10.3168/jds.S0022-0302(90)79005-4 2273146

[16] Welch JG. Rumination, Particle Size and Passage from the Rumen. J Anim Sci. Oxford University Press; 1982;54: 885–894. 10.2527/jas1982.544885x

[17] Tafaj M, Steingass H, Susenbeth A, Lang GU, Drochner W. [Effect of hay particle size at different concentrations and feeding levels on digestive processes and feed intake in ruminants. 1. Chewing activity and fermentation in the rumen]. Arch Anim Nutr. 1999;52: 167–84. 10.1080/17450399909386160 10548969

[18] Kaske M, Hatiboglu S, Engelhardt W V. The influence of density and size of particles on rumination and passage from the reticulo-rumen of sheep. Br J Nutr. Cambridge University Press; 1992;67: 235 10.1079/BJN19920027 1317720

[19] Kammes KL, Allen MS. Rates of particle size reduction and passage are faster for legume compared with cool-season grass, resulting in lower rumen fill and less effective fiber. J Dairy Sci. 2012;95: 3288–3297. 10.3168/jds.2011-5022 22612962

[20] Yeoman CJ, White BA. Gastrointestinal tract microbiota and probiotics in production animals. Ann Rev Anim Biosci. Annual Reviews; 2014;2: 469–486. 10.1146/annurev-animal-022513-114149 25384152

[21] Beauchemin KA. Ingestion and Mastication of Feed by Dairy Cattle. Vet Clin North Am Food Anim Pract. Elsevier; 1991;7: 439–463. 10.1016/S0749-0720(15)30794-5 1654175

[22] Jaster EH, Murphy MR. Effects of varying particle size of forage on digestion and chewing behavior of dairy heifers. J Dairy Sci. 1983;66: 802–810. 10.3168/jds.S0022-0302(83)81860-8 6304163

[23] Zali SM, Teimouri YA, Jafari SA. Effect of particle size and fragility of corn silage and alfalfa hay on intake, digestibility, performance, and chewing activity of fattening male lambs. Res Rev J Veternary Sci. Research and Reviews; 2015;1: 47–57.

[24] Beauchemin KA, Eriksen L, Nørgaard P, Rode LM. Short communication: Salivary secretion during meals in lactating dairy cattle. J Dairy Sci. 2008;91: 2077–2081. 10.3168/jds.2007-0726 18420637

[25] Hernández J, Benedito JL, Abuelo A, Castillo C. Ruminal acidosis in feedlot: from aetiology to prevention. ScientificWorldJournal. Hindawi; 2014;2014: 702572 10.1155/2014/702572 25489604 PMC4247954

[26] Helm JF, Dodds WJ, Hogan WJ, Soergel KH, Egide MS, Wood CM. Acid Neutralizing Capacity of Human Saliva. Gastroenterology. Elsevier; 1982;83: 69–74. 10.1016/S0016-5085(82)80286-2 7075945

[27] Izumi K, Okishio Y, Nagao N, Niwa C, Yamamoto S, Toda T. Effects of particle size on anaerobic digestion of food waste. Int Biodeterior Biodegradation. Elsevier; 2010;64: 601–608. 10.1016/J.IBIOD.2010.06.013

[28] Russell JB, Diez-Gonzalez F. The effects of fermentation acids on bacterial growth. Adv Microb Physiol. 1998;39: 205–234. 9328648 10.1016/s0065-2911(08)60017-x

[29] Russell JB, Wilson DB. Why are ruminal cellulolytic bacteria unable to gigest cellulose at low pH? J Dairy Sci. 1996;79: 1503–1509. 10.3168/jds.S0022-0302(96)76510-4 8880476

[30] Moreira LM, Leonel F de P, Vieira RAM, Pereira JC. A new approach about the digestion of fibers by ruminants. Rev Bras Saúde e Produção Anim. UFBA—Universidade Federal da Bahia; 2013;14: 382–395. 10.1590/S1519-99402013000200008

[31] Dhingra D, Michael M, Rajput H, Patil RT. Dietary fibre in foods: a review. J Food Sci Technol. Springer; 2012;49: 255–66. 10.1007/s13197-011-0365-5 23729846 PMC3614039

[32] Hironaka R, Kozub GC, Kimura N. Influence of feed particle size on rate and efficiency of gain, characteristics of rumen fluid and rumen epithelium, and numbers of rumen protozoa. Can J Anim Sci. 1979;59: 395–402. 10.4141/cjas79-049

[33] Xu M, Dong Y, Du S, Hao YS, Wang YH, Wang FN, et al Effect of corn particle size on mucosal morphology and digesta pH of the gastrointestinal tract in growing goats. Livest Sci. 2009;123: 34–37. 10.1016/j.livsci.2008.10.003

[34] Suarez FX. Particle size of calf starter: Effects on digestive system development and rumen fermentation. Pennsylvania State University. 2014.

[35] Norouzian MA, Valizadeh R. Effect of forage inclusion and particle size in diets of neonatal lambs on performance and rumen development. J Anim Physiol Anim Nutr (Berl). 2014;98: 1095–1101. 10.1111/jpn.12183 24661507

[36] Norouzian MA, Valizadeh R, Vahmani P. Rumen development and growth of Balouchi lambs offered alfalfa hay pre- and post-weaning. Trop Anim Heal Prod. 2011;43: 1169–1174. 10.1007/s11250-011-9819-z 21465104

[37] Van Soest PJ. Nutritional ecology of the ruminant. 2nd ed Ithaca: Cornell University Press; 1982.

[38] Dehority BA, Johnson RR. Effect of particle size upon the in vitro cellulose digestibility of forages by rumen bacteria. J Dairy Sci. 1961;44: 2242–2249. 10.3168/jds.S0022-0302(61)90051-0

[39] Al-Saiady MY, Abouheif MA, Makkawi AA, Ibrahim HA, Al-Owaimer AN. Impact of particle length of alfalfa hay in the diet of growing lambs on performance, digestion and carcass characteristics. Asian Aus J Anim Sci. 2010;23: 475–482.

[40] Karimizadeh E, Chaji M, Mohammadabadi T. The effects of physical form of diet on nutrient digestibility, rumen fermentation, rumination, growth performance and protozoa population of finishing lambs. Anim Nutr. 2017; 10.1016/j.aninu.2017.01.004 29767114 PMC5941103

[41] Belyea RL, Martz FA, Mbagaya GA. Effect of particle size of alfalfa hay on intake, digestibility, milk yield, and ruminal cell wall of dairy cattle. J Dairy Sci. 1989;72: 958–963. 10.3168/jds.S0022-0302(89)79189-X 2745816

[42] Hogue DE. Intake and fermentation rates of diets in growing lambs. Cornell Nutrition Conference. Ithaca, NY: Cornell University Agricultural Experiment Station; 1991. p. 83.

[43] Keady TWJ, Hanrahan JP. Effects of silage from maize crops differing in maturity at harvest, grass silage feed value and concentrate feed level on performance of finishing lambs. Animal. 2013;7: 1088–1098. 10.1017/S1751731113000104 23425683

[44] Vanzant ES, Cochran RC, Titgemeyer EC. Standardization of in situ techniques for ruminant feedstuff evaluation. J Anim Sci. 1998;76: 2717–2729. 10.2527/1998.76102717x 9814915

[45] Bourget SJ, Kemp JG. Wet seiving apparatus for stability analysis of soil aggregates. Can J Soil Sci. NRC Research Press Ottawa, Canada; 1957;37: 60–61. 10.4141/cjss57-009

[46] Van Dyne GM, Meyer JH. A method for measurement of forage intake of grazing livetsock using microdigestion techniques. J Range Manag Arch. Society for Range Management; 1964;17: 204–208.

[47] Package Lenth R. “lsmeans” Title Least-Squares Means. 2018 p. 5 10.1080/00031305.1980.10483031

[48] Ishaq SL, Johnson SP, Miller ZJ, Lehnhoff EA, Olivo S, Yeoman CJ, et al Impact of cropping systems, soil inoculum, and plant species identity on soil bacterial community structure. Microb Ecol. Microbial Ecology; 2017;73: 417–434. 10.1007/s00248-016-0861-2 27677892

[49] Callahan BJ, McMurdie PJ, Rosen MJ, Han AW, Johnson AJA, Holmes SP. DADA2: High-resolution sample inference from Illumina amplicon data. Nat Methods. Nature Publishing Group; 2016;13: 581–583. 10.1038/nmeth.3869 27214047 PMC4927377

[50] RCoreTeam. R: a language and environment for statistical computing. Vienna, Austria: R Foundation for Statistical Computing; 2018.

[51] Yeoman CJ, Ishaq SL, Bichi E, Olivo SK, Lowe J, Aldridge BM. Biogeographical differences in the influence of maternal microbial sources on the early successional development of the bovine neonatal gastrointestinal tract. Sci Rep. Springer US; 2018;8: 3197 10.1038/s41598-018-21440-8 29453364 PMC5816665

[52] McMurdie PJ, Holmes S. phyloseq: An R Package for Reproducible Interactive Analysis and Graphics of Microbiome Census Data. Watson M, editor. PLoS One. Public Library of Science; 2013;8: e61217 10.1371/journal.pone.0061217 23630581 PMC3632530

[53] Love MI, Huber W, Anders S. Moderated estimation of fold change and dispersion for RNA-seq data with DESeq2. Genome Biol. BioMed Central; 2014;15: 550 10.1186/s13059-014-0550-8 25516281 PMC4302049

[54] Archer E. Estimate Permutation p-Values for Random Forest ImportanceMetrics [R package rfPermute version 2.1.6]. Comprehensive R Archive Network (CRAN); 2018.

[55] Wickham H. ggplot2: Elegant graphics for Data Analysis. 2nd ed Springer Publishing Company; 2009.

[56] Bates D, Mächler M, Zurich E, Bolker BM, Walker SC. Fitting Linear Mixed-Effects Models Using lme4. 2015 10.18637/jss.v067.i01

[57] Oksanen J, Blanchet FG, Friendly M, Kindt R, Legendre P, Mcglinn D, et al Community Ecology Package. 2018.

[58] Mertens DR. Creating a system for meeting the fiber requirements of dairy cows. J Dairy Sci. 1997;80: 1463–1481. 10.3168/jds.S0022-0302(97)76075-2 9241608

[59] Merck Veterinary Manual [Internet]. 2019 [cited 31 Mar 2019]. Available: https://www.merckvetmanual.com/

[60] Huws SA, Mayorga OL, Theodorou MK, Kim EJ, Cookson AH, Newbold CJ, et al Differential colonization of plant parts by the rumen microbiota is likely to be due to different forage chemistries. J Microb Biochem Technol. OMICS International; 2014;6: 80–86. 10.4172/1948-5948.1000126

[61] Committee on Nutrient Requirements of Small Ruminants. Nutrient requirements of small ruminants: sheep, goats, cervids and New World camelids. 1st ed Washington: National Academies Press; 2007 10.17226/11654

[62] Colucci PE, Chase LE, Van Soest PJ. Feed Intake, Apparent Diet Digestibility, and Rate of Particulate Passage in Dairy Cattle. J Dairy Sci. Elsevier; 1982;65: 1445–1456. 10.3168/JDS.S0022-0302(82)82367-9

[63] Martz FA, Belyea RL. Role of particle size and forage quality in digestion and passage by cattle and sheep. J Dairy Sci. Elsevier; 1986;69: 1996–2008. 10.3168/jds.S0022-0302(86)80626-9 3745592

[64] Yang WZ, Beauchemin KA, Rode LM. Effects of Particle Size of Alfalfa-Based Dairy Cow Diets on Site and Extent of Digestion. J Dairy Sci. 2002;85: 1958–1968. 10.3168/jds.S0022-0302(02)74272-0 12214988

[65] Rode LM, Weakley DC, Sattler LD. Effect of forage amount and particle size in diets of lactating dairy cows on site of digestion and microbial protein synthesis. Can J Anim Sci. 1985;65: 101–111.

[66] Monod J. The Growth of Bacterial Cultures. Annu Rev Microbiol. Annual Reviews 4139 El Camino Way, P.O. Box 10139, Palo Alto, CA 94303–0139, USA; 1949;3: 371–394. 10.1146/annurev.mi.03.100149.002103

[67] Janssen PH. Influence of hydrogen on rumen methane formation and fermentation balances through microbial growth kinetics and fermentation thermodynamics. Anim Feed Sci Technol. Elsevier; 2010;160: 1–22. 10.1016/J.ANIFEEDSCI.2010.07.002

[68] Udén P. The effect of grinding and pelleting hay on digestibility, fermentation rate, digesta passage and rumen and faecal particle size in cows. Anim Feed Sci Technol. Elsevier; 1988;19: 145–157. 10.1016/0377-8401(88)90063-6

[69] Boyd JW. Creatine phosphokinase in normal sheep and in sheep with nutritional muscular dystrophy. J Comp Pathol. W.B. Saunders; 1976;86: 23–28. 10.1016/0021-9975(76)90023-2 1254743

[70] Gooneratne SR, Olkowski AA, Christensen DA. Sulfur-induced Polioencephalomalacia in Sheep: Some Biochemical Changes. PMC1255577 2574073

[71] Sekin S, Yoyvoda H, Bildik A, Yur F. The Importance of Serum Creatine Kinase (CK), Aspartate Aminotransferase (AST) and Lactate Dehydrogenase (LDH) Activities in the Diagnosis and Prognosis of Subclinic and Clinic White Muscle Disease in Lambs. Turkish J Vet Anim Sci. 1996;20: 225–230.

[72] Gooneratne SR, Howell JM. Creatine kinase release and muscle changes in chronic copper poisoning in sheep. Res Vet Sci. 1980;28: 351–61. 7414089

[73] Kock MD, Clark RK, Franti CE, Jessup DA. Effects of capture on biological parameters in free-ranging bighorn sheep (ovis canadensis): evaluation of normal, stressed and mortality outcomes and documentation of postcapture survival. J Wildl Dis. 1987;23: 652–662. 10.7589/0090-3558-23.4.652 3682092

[74] Hammond AC. Update on BUN and MUN as a guide for protein supplementation in cattle. Florida Ruminant Nutrition Symposium. 1997.

[75] Wang M, Zhao XG, Liao HY, Tan ZL, Tang SX, Sun ZH, et al Effects of rice straw particle size on digesta particle size distribution, nitrogen metabolism, blood biochemical parameters, microbial amino acid composition and intestinal amino acid digestibility in goats. Anim Sci J. 2011;82: 78–85. 10.1111/j.1740-0929.2010.00809.x 21269364

[76] Balish E, Yale CE, Hong R. Serum proteins of gnotobioic rats. Infect Immun. 1972;6: 112–118. 4120245 10.1128/iai.6.2.112-118.1972 PMC422502

[77] Bomba A, Žitnan R, Koniarová I, Lauková A, Sommer A, Pošivák J, et al Rumen fermentation and metabolic profile in conventional and gnotobiotic lambs. Arch für Tierernaehrung. Taylor & Francis Group; 1995;48: 231–243. 10.1080/17450399509381844 8585797

[78] Wikoff WR, Anfora AT, Liu J, Schultz PG, Lesley SA, Peters EC, et al Metabolomics analysis reveals large effects of gut microflora on mammalian blood metabolites. Proc Natl Acad Sci U S A. National Academy of Sciences; 2009;106: 3698–703. 10.1073/pnas.0812874106 19234110 PMC2656143

[79] Soares JH, Leffel EC, Larsen RK. Neonatal lambs in a gnotobiotic environment. J Anim Sci. The American Society of Animal Science; 1970;31: 733 10.2527/jas1970.314733x 5455686

[80] Khalid MF, Shahzad MA, Sarwar M, Rehman AU, Sharif M, Mukhtar N. Probiotics and lamb performance: a review. African J od Agric Res. 2011;6: 5198–5203.

