# Supplementary material for: Pelleted-hay alfalfa feed increases sheep wether weight gain and rumen bacterial richness over loose-hay alfalfa feed
Source: PLoS One. 2019 Jun 5;14(6):e0215797. doi: 10.1371/journal.pone.0215797 (PMC6550389; doi:10.1371/journal.pone.0215797)
Supplement: S1 Table — (DOCX) [file pone.0215797.s001.docx]

**S1 Table Serum parameters for wethers receiving either a loose-hay or a pelleted-hay alfalfa diet treatment for two weeks.**

| Pelleted-Hay Alfalfa | Week 2 | SD | 34.4 | 12.7 | 11.2 | 63.7 | 6.6 | 0.2 | 0.2 | 0.3 | 0.2 | 0.7 | 2.6 | 0.1 | 0 | 0 | 1.4 | 0.4 | 0.9 | 2.9 | 0.1 | 0.1 |
| --- | --- | --- | --- | --- | --- | --- | --- | --- | --- | --- | --- | --- | --- | --- | --- | --- | --- | --- | --- | --- | --- | --- |
|  |  | Mean | 243.8 | 112.8 | 77.0 | 214.2 | 66.2 | 6.9 | 3.0 | 3.9 | 10.4 | 5.7 | 31.4 | 0.8 | 0.3 | 0.1 | 143 | 4.4 | 104.4 | 24.8 | 2.4 | 0.2 |
|  | Week 1 | SD | 263.5 | 15.8 | 9.9 | 52.7 | 8.3 | 0.2 | 0.2 | 0.3 | 0.2 | 0.6 | 3.6 | 0.1 | 0.1 | 0 | 1.3 | 0.5 | 1.1 | 1.7 | 0.1 | 0.1 |
|  |  | Mean | 666.8 | 120.2 | 80.4 | 203.6 | 62 | 6.9 | 3.2 | 3.7 | 10.9 | 6.3 | 32.2 | 0.9 | 0.5 | 0.1 | 142.8 | 4.9 | 105.2 | 24.1 | 2.5 | 0.2 |
|  | Week 0 | SD | 375.6 | 12.1 | 14.8 | 60.3 | 9 | 0.2 | 0.1 | 0.1 | 0.3 | 0.6 | 2.1 | 0.1 | 0.1 | 0 | 1.5 | 0.3 | 1.6 | 2.7 | 0.1 | 0.1 |
|  |  | Mean | 456.2 | 117 | 79.2 | 205.8 | 72.4 | 6.7 | 3.2 | 3.5 | 10.4 | 6.6 | 32.6 | 0.8 | 0.4 | 0.1 | 143.4 | 5.0 | 104 | 22.8 | 2.4 | 0.2 |
|  |  |  |  |  |  |  |  |  |  |  |  |  |  |  |  |  |  |  |  |  |  |  |
| Loose-Hay Alfalfa | Week 2 | SD | 36.3 | 21.1 | 22 | 18.8 | 4.2 | 0.5 | 0.1 | 0.5 | 0.3 | 0.4 | 3.7 | 0.2 | 0.1 | 0.1 | 0.7 | 0.3 | 0.8 | 3.3 | 0.1 | 0.2 |
|  |  | Mean | 241.4 | 112.4 | 85.6 | 221.2 | 63.0 | 6.7 | 3.1 | 3.6 | 10.2 | 7.6 | 26.4 | 0.7 | 0.3 | 0.1 | 144 | 4.6 | 104.2 | 27.6 | 2.3 | 0.3 |
|  | Week 1 | SD | 26.2 | 23.3 | 19 | 26.6 | 10.5 | 0.4 | 0.1 | 0.3 | 0.3 | 1.4 | 2.7 | 0.1 | 0.1 | 0 | 0.8 | 0.1 | 0.8 | 2.8 | 0.2 | 0.1 |
|  |  | Mean | 314.8 | 120.6 | 84.6 | 215.0 | 64.6 | 6.5 | 3.1 | 3.4 | 10.3 | 8.1 | 26.6 | 0.7 | 0.5 | 0.1 | 143.8 | 4.9 | 104.8 | 27.2 | 2.3 | 0.3 |
|  | Week 0 | SD | 464.9 | 36.7 | 19.8 | 50.3 | 10.1 | 0.4 | 0.2 | 0.3 | 0.3 | 0.8 | 3.1 | 0.1 | 0 | 0.1 | 3.8 | 0.4 | 3.5 | 2.1 | 0.2 | 0.1 |
|  |  | Mean | 516.6 | 130.8 | 85.2 | 247.4 | 75.4 | 6.5 | 3.2 | 3.3 | 10.4 | 6.6 | 31.8 | 0.7 | 0.4 | 0.1 | 144.2 | 4.7 | 105 | 23.6 | 2.3 | 0.3 |
|  |  |  | CPK | AST | FFT | ALKp | Glucose | Total Protein | Albumin | Globulin | Calcium | Phosphorus | BUN | Creatinine | Total Bilirubin | Direct Bilirubin | Sodium | Potassium | Chlorine | Total carbon dioxide | Magnesium | Hemolysis Index |

The panel included creatine kinase/phosphokinase (CPK), creatinine (CREAT), aspartate aminotransferase (AST), alkaline phosphatase (ALKP), total protein (TP), albumin (ALB), globulin (GLOB), glucose (GLU), blood urea nitrogen (BUN), calcium (Ca), phosphorus (P), sodium (Na), potassium (K), chloride (Cl), magnesium (Mg), total bilirubin (T Bili), direct bilirubin (D Bili), and total carbon dioxide (TCO2), as well as Icteric index and Lipemic index which were both 0.17 for all wethers.
