# Supplementary material for: Pelleted-hay alfalfa feed increases sheep wether weight gain and rumen bacterial richness over loose-hay alfalfa feed
Source: PLoS One. 2019 Jun 5;14(6):e0215797. doi: 10.1371/journal.pone.0215797 (PMC6550389; doi:10.1371/journal.pone.0215797)
Supplement: S2 Table — (DOCX) [file pone.0215797.s002.docx]

**S2 Table Residual nutritional composition of feces from wethers on loose-hay or pelleted-hay alfalfa diets over the two-week experimental period.**

| **Day** | **Moisture** | | **DM (%)** | | **CP (%DM)** | | **ADF (%DM)** | | **NDF (%DM)** | | **Lignin** | |
| --- | --- | --- | --- | --- | --- | --- | --- | --- | --- | --- | --- | --- |
|  | **Mean** | **SD** | **Mean** | **SD** | **Mean** | **SD** | **Mean** | **SD** | **Mean** | **SD** | **Mean** | **SD** |
| **Loose-Hay Alfalfa** | | | | | | | | | | | |  |
| **0-2** | 1.7 | 0.4 | 98.3 | 0.3 | 15.5 | 0.8 | 44.3 | 5.7 | 57.6 | 3.4 | 17.5 | 2.5 |
| **3-6** | 1.5 | 0.4 | 98.5 | 0.4 | 16.6 | 0.7 | 41.2 | 2.9 | 53.3 | 3.2 | 17.1 | 1.2 |
| **7-10** | 1.6 | 0.7 | 98.4 | 0.7 | 15.3 | 0.3 | 42.2 | 5.0 | 54.8 | 3.5 | 16.9 | 2.6 |
| **11-14** | 1.4 | 0.3 | 98.7 | 0.3 | 14.8 | 0.8 | 41.4 | 2.9 | 55.8 | 1.4 | 16.4 | 1.3 |
| **Pelleted-Hay Alfalfa** | | | | | | | | | | | |  |
| **0-2** | 1.4 | 0.8 | 98.6 | 0.8 | 14.7 | 0.6 | 43.5 | 3.7 | 58.4 | 2.5 | 14.8 | 1.2 |
| **3-6** | 1.0 | 0.5 | 99.0 | 0.5 | 14.3 | 0.5 | 45.8 | 3.0 | 59.5 | 2.0 | 16.0 | 1.9 |
| **7-10** | 1.0 | 0.4 | 99.0 | 0.4 | 14.4 | 0.4 | 44.2 | 1.9 | 55.4 | 0.8 | 15.9 | 1.3 |
| **11-14** | 0.9 | 0,4 | 99.1 | 0.3 | 13.8 | 0.7 | 44.1 | 2.7 | 58.2 | 3.9 | 15.9 | 1.7 |

Crude protein (% DM) was significantly different by diet (lmer, *p* = 0.00150) and day (lmer, *p* = 0.00285) overall.
