# Supplementary material for: Pelleted-hay alfalfa feed increases sheep wether weight gain and rumen bacterial richness over loose-hay alfalfa feed
Source: PLoS One. 2019 Jun 5;14(6):e0215797. doi: 10.1371/journal.pone.0215797 (PMC6550389; doi:10.1371/journal.pone.0215797)
Supplement: S3 Table — (DOCX) [file pone.0215797.s003.docx]

**S3 Table Daily intake by diet.**

| **Day** | | **Hay (kg)** | **Pellet (kg)** |
| --- | --- | --- | --- |
| 0 | 1.90 | | 2.29 |
| 1 | 1.72 | | 1.93 |
| 2 | 1.55 | | 1.63 |
| 3 | 1.76 | | 1.78 |
| 4 | 1.26 | | 1.87 |
| 5 | 1.57 | | 1.89 |
| 6 | 1.22 | | 0.65 |
| 7 | 3.17 | | 2.78 |
| 8 | 1.61 | | 2.05 |
| 9 | 1.47 | | 2.01 |
| 10 | 1.78 | | 2.03 |
| 11 | 1.45 | | 2.12 |
| 12 | 1.17 | | 2.05 |
| 13 | 0.66 | | 0.95 |
| 14 | NA | | NA |
